# Viruses and Phytoparasitic Nematodes of *Cicer arietinum* L.: Biotechnological Approaches in Interaction Studies and for Sustainable Control

**DOI:** 10.3389/fpls.2018.00319

**Published:** 2018-03-15

**Authors:** Paola Leonetti, Gian Paolo Accotto, Moemen S. Hanafy, Vitantonio Pantaleo

**Affiliations:** ^1^Institute for Sustainable Plant Protection, Research Unit of Bari, National Research Council, Bari, Italy; ^2^Institute for Sustainable Plant Protection, Research Unit of Turin, National Research Council, Turin, Italy; ^3^Department of Plant Biotechnology, National Research Centre, Cairo, Egypt

**Keywords:** *Cicer arietinum* L., plant viruses, plant parasitic nematodes, RNA silencing, viral metagenomics, plant transformation, genome editing

## Abstract

*Cicer arietinum* L. (chickpea) is the world's fourth most widely grown pulse. Chickpea seeds are a primary source of dietary protein for humans, and chickpea cultivation contributes to biological nitrogen fixation in the soil, given its symbiotic relationship with rhizobia. Therefore, chickpea cultivation plays a pivotal role in innovative sustainable models of agro-ecosystems inserted in crop rotation in arid and semi-arid environments for soil improvement and the reduction of chemical inputs. Indeed, the arid and semi-arid tropical zones of Africa and Asia have been primary areas of cultivation and diversification. Yet, nowadays, chickpea is gaining prominence in Canada, Australia, and South America where it constitutes a main ingredient in vegetarian and vegan diets. Viruses and plant parasitic nematodes (PPNs) have been considered to be of minor and local impact in primary areas of cultivation. However, the introduction of chickpea in new environments exposes the crop to these biotic stresses, compromising its yields. The adoption of high-throughput genomic technologies, including genome and transcriptome sequencing projects by the chickpea research community, has provided major insights into genome evolution as well as genomic architecture and domestication. This review summarizes the major viruses and PPNs that affect chickpea cultivation worldwide. We also present an overview of the current state of chickpea genomics. Accordingly, we explore the opportunities that genomics, post-genomics and novel editing biotechnologies are offering in order to understand chickpea diseases and stress tolerance and to design innovative control strategies.

## *Cicer arietinum* L.: uses, origin, and distribution

In many developing countries, grain legumes have gained much importance in view of acute shortages in the production of animal proteins and the prevalence of protein malnutrition. Conversely, they are a valid alternative as a source of protein for specific (vegetarian or vegan) or balanced diets worldwide, particularly in developed countries.

Legumes are able to fix atmospheric nitrogen, in association with bacteria, and play a central role in low-input and sustainable agricultural systems (Graham and Vance, 2003). With a global production of ca. 77 × 10^6^ tons, grain legumes (also known as “pulses”) rank third after cereals and oilseeds (FAO, 2014). The world production of chickpea in 2014 was more than 13 × 10^6^ tons (FAO, 2014), making chickpea rank fourth among the pulses after soybean, peanut, and common bean. However, chickpea can be considered the most important crop at regional level, especially in semi-arid areas of the world and in Mediterranean regions (FAO, 2014). The genus *Cicer* L. includes 44 taxa, 9 annuals, and 35 perennials, and has a narrow genetic base, probably as a consequence of it being a monophyletic descendent from its wild progenitor *Cicer reticulatum*, grown in the Fertile Crescent region (the center of chickpea domestication and diversification) (Abbo et al., 2003). The most popularly known species is the cultivated *Cicer arietinum* L., with 2*n* = 2*x* = 16 chromosomes and a genome size of ~738 Mb (Varshney et al., 2013). Commercially, the cultivated chickpea varieties are grouped according to the plant's flowers pigmentation as well as size and color of seeds; i.e., *desi*-type (small-seeded) and *kabuli*-type (large-seeded). *Desi*-type accounts for about 85% of the world's production and is mainly grown in India, Pakistan, Iran, Afghanistan, and Ethiopia. *kabuli*-type, instead, is grown in the Middle East, India, Mexico, North and South America, Australia, Spain, and Italy. A third type is characterized by a medium-to-small size and cream-colored seed, and it is designated as “pea-shaped” (Upadhyaya et al., 2008). Seed color (black, red, or white, and their variations) is a key commercial characteristic, which is also associated with the content of phenylpropanoid pathway-derived bioactive secondary metabolites such as flavonoids, lignans, and isoflavones. In addition to seed coat color determination, these secondary products have potential medicinal properties (Sirtori, 2001), and varied and important functions in processes, such as UV protection, disease resistance, and nodulation (N_2_ fixation) (Reinprecht et al., 2013).

The *ex-situ* collections of chickpea landraces and wild relatives are stored in 44 genebanks worldwide (Smýkal, 2015) and hold a combination of 98,313 accessions. The largest collections are conserved at the International Crops Research Institute for the Semi-Arid Tropics (ICRISAT) in India (20,140 accessions) and International Center for Agricultural Research in the Dry Areas (ICARDA) in Syria (13,818 accessions) (Table 1). Chickpea underwent a drastic loss of genetic diversity due to a series of bottlenecks unique to this crop, i.e., (i) reluctant cross-compatibility with wild species, (ii) difficulty in domestication, and (iii) winter-spring annual phenology (Abbo et al., 2003). Consequently, *C. arietinum* displays a lack of adaptive diversity for a range of biotic and abiotic stress. Susceptibility to viruses, pathogens and pests, sensitivity to environmental stress and poor cross-pollination are the main reasons for the limited diffusion and low production of chickpea.

**Table 1:** Major “depositor institutes” conserving chickpea accessions.

**Genebank and link**	**Acronym**	**Country**	**Accessions**
International Crop Research Institute for the Semi-Arid Tropics http://www.icrisat.org	ICRISAT	India	20,140
International Center for Agricultural Research in the Dry Areas http://www.icarda.org	ICARDA	Syria	13,818
United States Department of Agriculture https://www.ars-grin.gov	USDA	USA	6,789
Aegean Agricultural Research Institute http://www.gfar.net	AARI	Turkey	2,075
Australian Temperate Field Crops Collection http://elibrary.grdc.com.au	GRDC	Australia	8,655
National Plant Gene Bank http://medomed.org	NPGB	Iran	5,700
Vavilov Institute of Plant Genetic Resources https://www.gbif.org	VIR	Russia	2,091
Institute for Agrobotany Tapi' oszele https://www.nebih.gov.hu	nèbih	Hungary	1,170
Mediterranean Germplasm Database http://ibbr.cnr.it/mgd/	MGR_IBBR_CNR	Italy	358

## Viruses and virus-like entities hosted by *C. arietinum* L.

**Figure 1:**
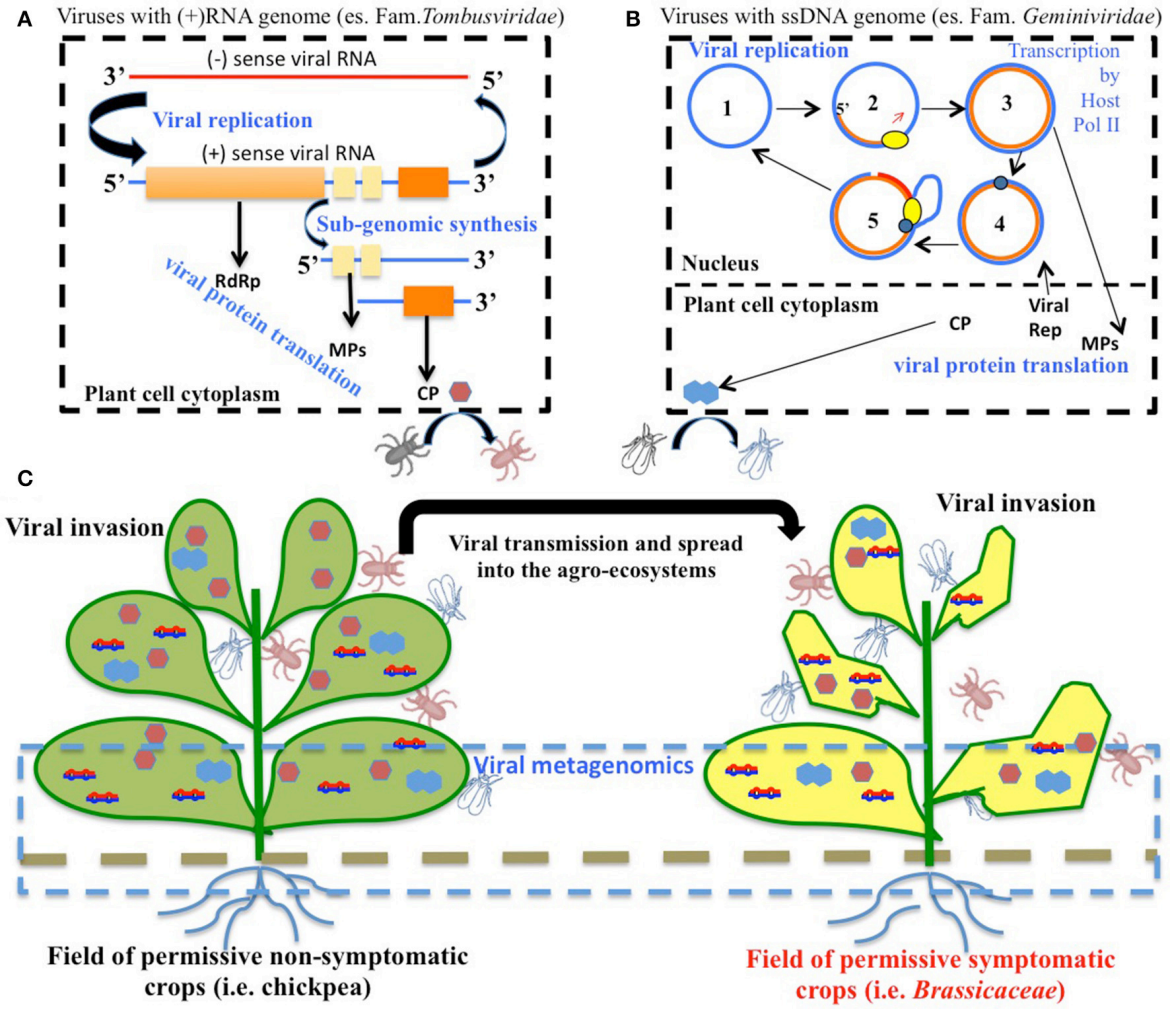
Virus cycles in agro-ecosystems. **(A)** Schematic representation of replication cycles of *Turnip crinkle virus* (TCV) family *Tombusviridae*, genus *Carmovirus*), a virus that has been found associated to chickpea in open field. TCV has a positive (+) sense RNA genome that replicates (blue line). The viral RNA-dependent RNA polymerase (RdRp) amplifies the viral genome in the cytoplasm via negative (−) sense RNA template synthesis (red line). (+) RNA enters into the cellular translation machinery and codes for the RdRp. Moreover, movement proteins (MPs) and coat protein (CP) are the products of translation on viral sub-genomic RNAs. TCV genomic RNA can be encapsidated by the CP to form an icosahedral virion that can be then acquired by insects such as coleoptera. **(B)** Schematic representation of replication cycles of single stranded (ss) DNA viruses of the family *Geminiviridae*. Circular viral genomic ssDNA (1) functions as template for the synthesis of antisense ssDNA (orange line) due to the activity of host DNA-dependent DNA polymerase (yellow element) (2) to form a viral double stranded (ds)DNA intermediate (3). Viral dsDNA can be transcribed in the nucleus by the host DNA-dependent RNA polymerase PolII. Viral RNA transcripts are transferred to the cytoplasm, and enter into the translational machinery to release viral replicase (rep, blue element), MP and CP. One strand of the viral dsDNA can undergo cleavage by viral rep (4), thus allowing the access to the host DNA polymerase that extends the viral ssDNA and generates several copies of the circular genome (5). The ssDNA can be encapsidated and acquired by leafhopper vectors. **(C)** Chickpea is a permissive, non-symptomatic host for several viruses and it is often used in rotation with and/or in proximity to other crops for a sustainable agriculture. It therefore functions as a reservoir of virus inoculum that can be spread through insect vectors to other permissive crops that can show viral symptoms such as leaf yellowing, curling deformation and a general impact on the crop production. Metagenomics of nucleic acids of viral origin can be applied on either symptomatic or non-symptomatic plant tissues, as well as to other environmental samples (soil, insects) in order to explore viral entities associated to agro-ecosystems.

Several viruses have been isolated from naturally infected chickpea worldwide, but only a few cause diseases, which under specific environmental conditions can lead to significant economic loss (Bos et al., 1988; Kumar et al., 2008). The most relevant viruses reported to infect and induce disease in chickpea are: Alfalfa mosaic virus (AMV, *Alfamovirus, Bromoviridae*), Cucumber mosaic virus (CMV, *Cucumovirus, Bromoviridae*), Bean leafroll virus (BLRV) and Beet western yellows virus (BWYV) (both *Luteovirus, Luteoviridae)*, Pea enation mosaic virus complex (PEMV-1, *Enamovirus, Luteoviridae*) and (PEMV-2, *Umbravirus*), Chickpea stunt disease-associated virus (CpSDaV, genus unassigned, *Luteoviridae*), and a number of geminiviruses of the genus *Mastrevirus*, the most important being Chickpea chlorotic dwarf virus (CpCDV). Faba bean necrotic yellows virus (FBNYV, *Nanovirus, Nanoviridae*) has also been reported (Makkouk et al., 2012). Table 2 contains a list of all the viruses to date associated with chickpea. Figure 1 contains a schematic representations of life cycles and spread of two groups of plant viruses included in Table 2 (i.e., with RNA or DNA genome).

**Table 2:** Viruses reported to infect chickpea.

**Family**	**Virus**	**Type of transmission**	**Countries**	**Genetic resistance**	**Some References**
*Bromoviridae*	Alfalfa mosaic virus (AMV, genus *Alfamovirus*)	Aphids (non-pers.), seeds, sap^*^	Iran	–	Kaiser and Danesh, 1971; Makkouk et al., 2003
	Cucumber mosaic virus (CMV, genus *Cucumovirus*)	Aphids (non-pers.), seeds, sap^*^	Iran, Morocco	–	Kaiser and Danesh, 1971; Ouizbouben and Fortass, 1997; Makkouk et al., 2003
*Luteoviridae*	Bean leafroll virus (BLRV, genus *Luteovirus*)	Aphid (pers.)	Azerbaijan, Iran, India, Turkey	–	Kaiser and Danesh, 1971
	Beet western yellows virus (BWYV, genus *Polerovirus*)	Aphids (pers.)	Azerbaijan, Iran	–	Makkouk et al., 2003; Mustafayev et al., 2011
	Soybean dwarf virus (SbDV, genus *Lutetovirus*)	Aphids	Iran, Syria	–	Makkouk et al., 2003; Kumari and Makkouk, 2007
	Chickpea chlorotic stunt virus (CpCSV, genus *Polerovirus*)	Aphids (pers.)	Ethiopia, Syria, Egypt, Eritrea, Iran, Morocco, Sudan	–	Abraham et al., 2006, 2009; Asaad et al., 2009; Banane et al., 2010
	Pea enation mosaic virus-1 (PEMV-1, genus *Enamovirus*)	Aphids (pers.), seeds, sap^*^	Canada, USA, Iran, Syria	yes	Makkouk et al., 2003
*Potyviridae*	Bean yellow mosaic virus (BYMV, genus *Potyvirus*)	Aphids (non-pers.), seeds, sap^*^	Iran, Algeria, Morocco	–	Makkouk et al., 2003
	Turnip mosaic virus (TuMV, genus Potyvirus)	Aphids (non-pers.), seeds, sap^*^	Australia	–	Schwinghamer et al., 2007
	Pea seed-borne mosaic virus (PSbMV, genus *Potyvirus*)	Aphid, seeds, sap^*^	Iran, Australia, Morocco, Algeria	yes	Ouizbouben and Fortass, 1997
*Nanoviridae*	Faba bean necrotic yellows virus (FBNYV, genus Nanovirus)	Aphids (pers.)	Jordan, Syria, Turkey, Lebanon, Iran, Egypt, Algeria	–	Yahia et al., 1997
*Geminiviridae*	Chickpea chlorotic dwarf virus (CpCDV, genus *Mastrevirus*)	Leafhoppers	India, Iran, Syria, Turkey	–	Horn et al., 1993; Makkouk et al., 2003
	Tobacco yellow dwarf virus (TYDV, genus *Geminiviridae*)	Leafhoppers	Australia	–	Thomas et al., 2010
*Virgaviridae*	Tomato mottle mosaic Virus (ToMMV, genus *Tobamovirus*)	Seeds, sap^*^	Italy	–	Pirovano et al., 2014
*Tombusviridae*	Turnip crinkle virus (TCV, genus *Carmovirus*)	Coleoptera, sap^*^	Italy	–	Ghasemzadeh et al., 2018
*Pospiviroidae*	Hop stunt viroid (HSVd, genus *Hostuviroid*)	Sap	Italy	–	Pirovano et al., 2014

*pers., persistent*;

* *Mechanical infection by wounding using infectious sap as inoculum*.

In recent years, the most invasive chickpea virus has been CpCDV. This virus, first reported in India in 1993 (Horn et al., 1993), has recently spread in many countries and among several crops, including other leguminous species (faba bean, lentil, bean), some solanaceous (tomato, pepper) and cucurbits (squash, cucumber), as well as other unrelated species such as cotton, sugar beet, okra, and papaya (Manzoor et al., 2014; Fahmy et al., 2015; Kraberger et al., 2015; Ouattara et al., 2017). In a newly discovered disease of watermelon in Tunisia, causing fruit hardness, CpCDV has been found as the causal agent (Zaagueri et al., 2017a,b). CpCDV is known to be transmitted by leafhopper species of the genus *Orosius* in a persistent manner (Horn et al., 1994). Today, CpCDV has attained a very wide distribution, including the Indian subcontinent, the Middle East and North Africa. Being so polyphagous and having a very widespread vector, CpCDV is certainly an emerging pest that will most likely colonize new areas (and possibly hosts) in forthcoming years.

In the last two decades, chickpea cultivation has been exposed to viral infections in novel areas of cultivation, such as Australia, where a high incidence of disease due to outbreak of viruses has been detected. The Australian food and agriculture stakeholders are closely observing chickpea cultivations and claiming the need to develop strategies that can assist in avoiding future viral epidemics in chickpea and other pulse crops. The Australian Grains Research and Development Corporation (GRDC) (Table 1) is supporting surveys of chickpea viruses in Central and West Asia (Kumari et al., 2011). As a result, other geminiviruses similar to CpCDV (but not CpCDV) have been found (Thomas et al., 2010; Hadfield et al., 2012), though currently limited to Australia.

Some chickpea viruses have a recognized quarantine significance, as tested by the Germplasm Health Laboratory of ICRISAT and ICARDA (see the Crop Genebank Knowledge Base website: https://cropgenebank.sgrp.cgiar.org/index.php/management-mainmenu-433/stogs-mainmenu-238/chickpea/guidelines/viruses). They are: Pea seed-borne mosaic virus, Bean yellow mosaic virus (PSbMV and BYMV, respectively; *Potyvirus, Potyviridae*), AMV and CMV (Table 2). Although belonging to different families, these viruses are transmitted by aphids and are also seed-transmitted to variable degrees. This last feature is of paramount importance for international trade, because viruses can reach and invade new habitats by the long distance human transport of seeds. Table 2 highlights the commercial sources of resistance against viruses, which to date are only two: PEMV-1 and PSbMV. For other viruses, no resistance has been described in the literature.

Recently, next-generation sequencing (NGS) approaches have opened the door to reconstructing viral populations in a high-throughput and cost-effective manner. Nowadays, NGS can be employed in environmental studies in the agro-ecosystem to either analyze known plant viruses by means of a reference-guided approach or to discover novel plant viruses using a *de novo*-based strategy (Massart et al., 2014).

**Figure 2:**
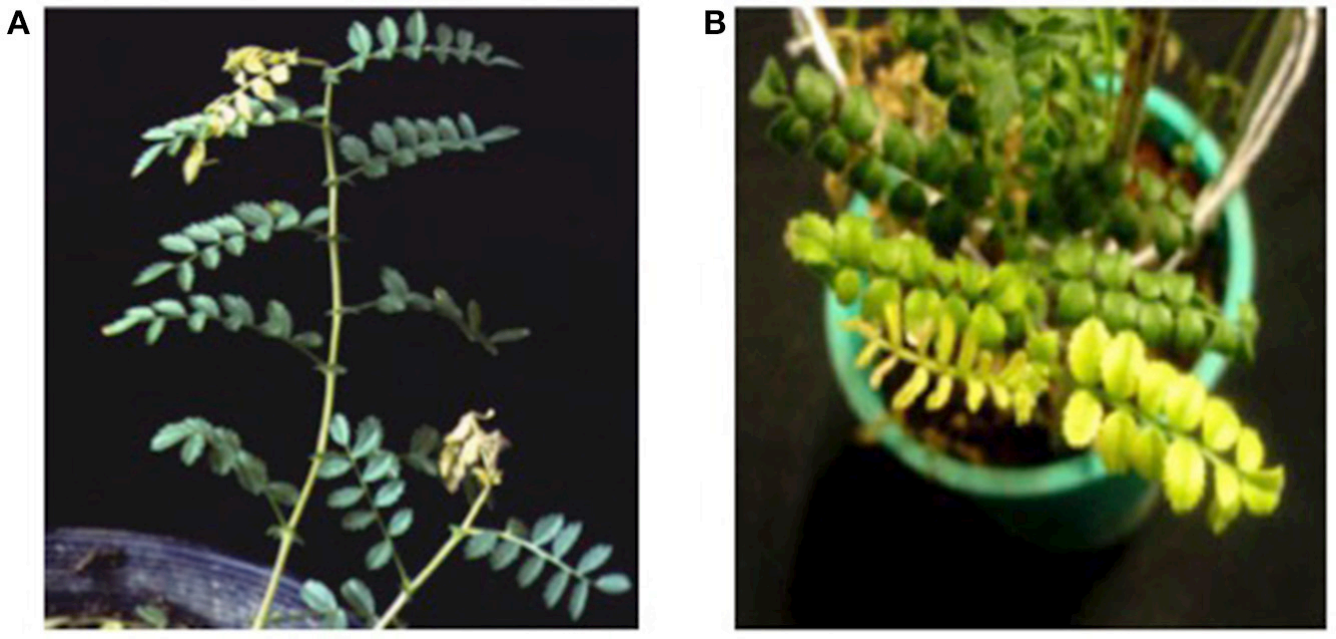
Selected photos showing symptoms induced by viruses on chickpea plants. **(A)** Tip wilting induced by mechanical inoculation with TuMV (from Schwinghamer et al., 2007). **(B)** Symptoms of chlorotic stunt disease caused by CpCDV on chickpea (from Kanakala et al., 2013).

Viral surveys using metagenomics in *C. arietinum* L. based on short (s)RNA analysis have been carried out in Apulia, Southern Italy, during the 2013–2017 time period. The surveys revealed that a large number of known viral species co-infect chickpea plants without causing any symptoms. Surprisingly, among the viruses found were Tomato mottle mosaic virus (ToMMV, *Tobamovirus, Virgaviridae*), which had not yet been observed in chickpea or reported in Europe, and one viroid referring to *Hop stunt viroid* (HSVd, *Hostuviroid, Pospiviroidae*) (Pirovano et al., 2014). In the same surveys, but in different plant samples, a high level of Turnip crinkle virus (TCV, *Carmovirus, Tombusviridae*) was found, though never reported before in chickpea (Ghasemzadeh et al., 2018). Worthy of note, viral metagenomics is showing that chickpea in open field is a highly permissive host for viruses and mixed infections are not uncommon. This means that most of the symptomatology that in the literature was ascribed to specific infections deserves further studies. In Figure 2 some viral symptoms that could be unequivocally ascribed to infection by a single virus.

To date, other virus-like infectious agents, such as phytoplasmas, have been reported only in sporadic cases, i.e., Australia, Ethiopia, Oman, and Pakistan. In most cases, phytoplasmas were associated with yellowing, phyllody and little leaves. Generally, infectious phytoplasmas are recognized as members of the 16SrII peanut witches' broom group (Ghanekar et al., 1988; Saqib et al., 2005; Al-Saady et al., 2006; Akhtar et al., 2008).

## PPNs associated with *C. arietinum* L.

**Figure 3:**
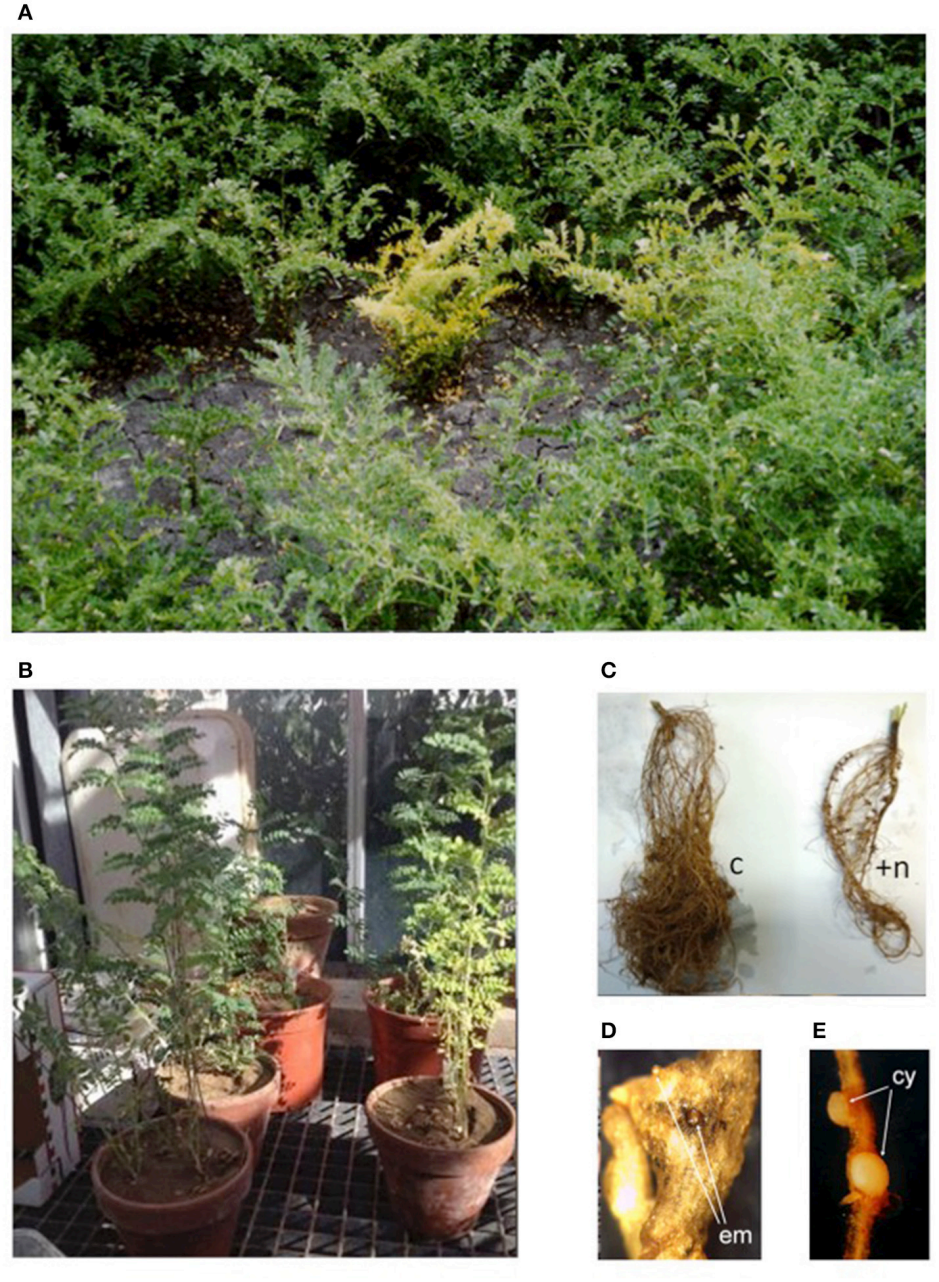
Symptoms of nematode infection on chickpea plants in field and greenhouse. **(A)** Symptoms of infection visible in the field on *C. arietinum*: plant reduced in crop yield with chlorotic, pale, and yellow leaves. **(B)** Greenhouse pot test: control plant (left) and *M. incognita* infected plant (right). **(C)** Root system of control (c) and *M. incognita* infected plants (+*n*). **(D)** Egg masses (em) generated by *M. incognita* mature female, in root galling tissue. **(E)** Newly formed cysts (cy) *of H. goettingiana*.

**Figure 4:**
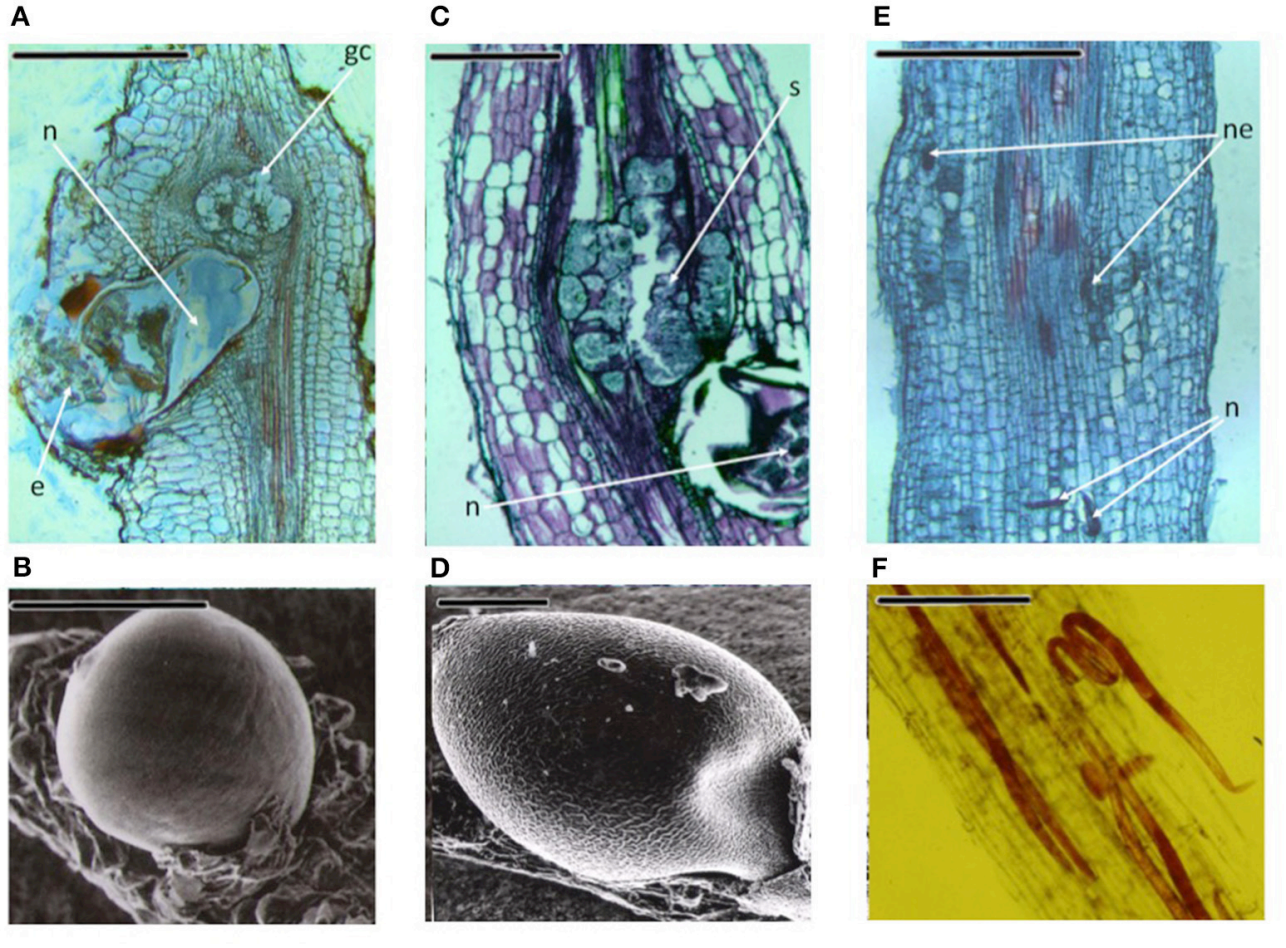
Three important PPNs associated to chickpea roots. *Meloidogyne artiellia*: **(A)** Longitudinal root section showing anatomical alterations; **(B)** Scanning electron microscopy (SEM) photo of a female on the root. *Heterodera ciceri*: **(C)** The tissues disruption caused by the cyst nematode is shown in longitudinal root section; (D) SEM image of a mature female. *Pratylenchus thornei*: **(E)** Longitudinal section of the root showing lesions caused by the nematode; **(F)** Fuchsin-stained root cortex section, showing the migratory endoparasite. n, nematode; e, eggs; gc, giant cell; ne, necrotic tissues; s, syncytium. Scale bars: **(A,C,E)** = 500 μm; **(B,D,F)** = 200 μm (Source: Nicola Vovlas, CNR).

**Figure 5:**
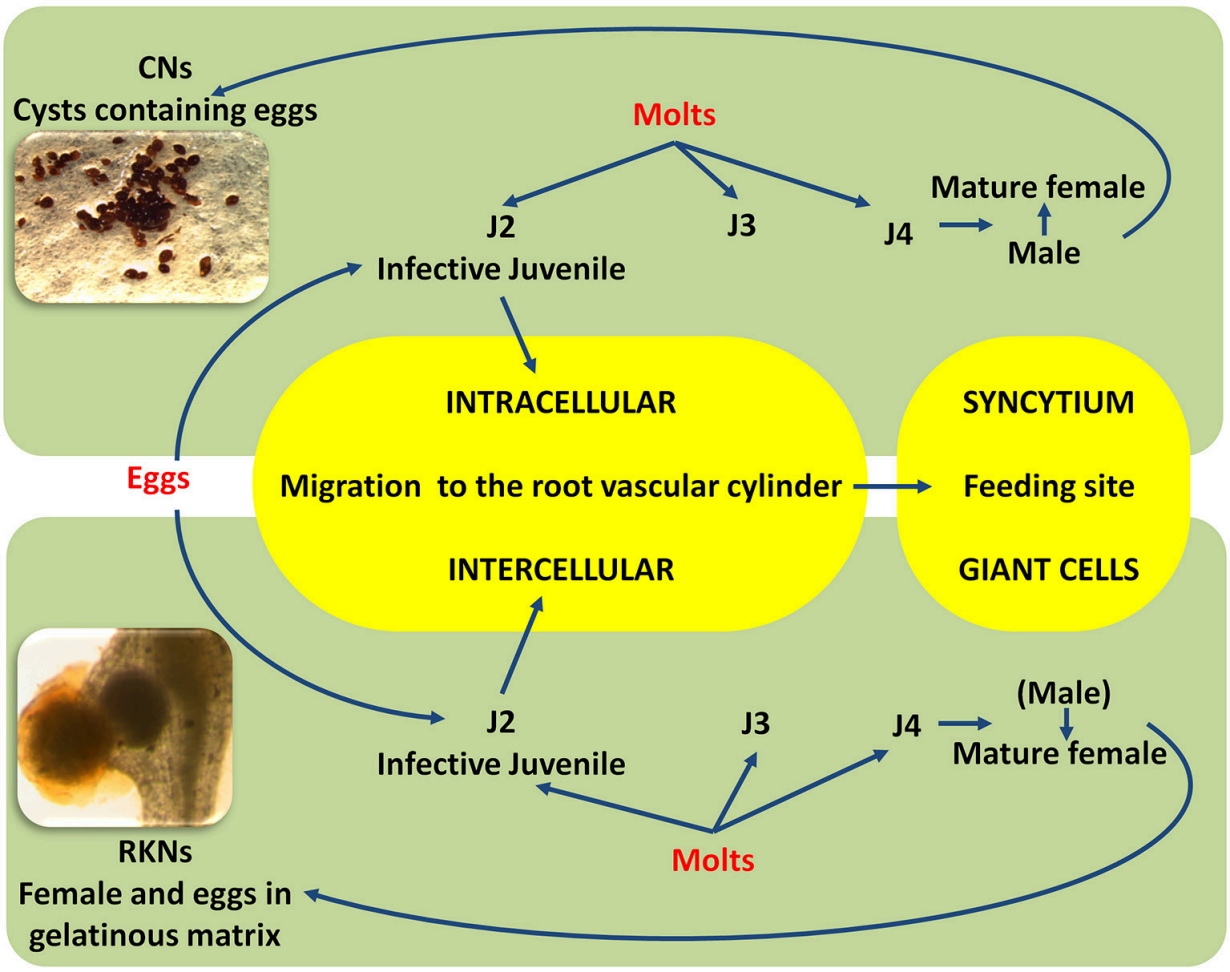
Simplified life cycles of cyst nematodes (CNs) and root-knot (RKNs) nematodes. Larvae hatch from cysts or from egg masses; the first-stage juvenile molts inside the eggshell become an invasive second-stage juvenile (J2) adapted to penetrate the root using an intra, inter-cellular migration and to the establishment of the feeding site (Syncytium and Giant cell). The nematode has to change molts (J3, J4) to become a fully mature (male or female) adult. Parthenogenetic and amphimictic reproduction modalities are different between CNs and RKNs.

Diseases caused by soil-borne PPNs can generate significant yield losses in economically relevant crops (De Coninck et al., 2015). The estimation of plant parasitic nematodes (PPN) constrains to chickpea production was estimated in 14% (Castillo et al., 2008). PPNs are biotrophic (i.e., obligate parasites that are completely dependent on the host as the only source of nutrients) and polyphagous, because they can infect many different hosts among monocots and dicots. In the most representative PPNs families, root-lesion nematodes (*Pratylenchus* spp.), reniform nematodes (*Rotylenchus reniformis*), cyst-forming nematodes (CNs) (*Heterodera* spp.) and root-knot nematodes (RKNs) (*Meloidogyne* spp.) have been found pathogenic for chickpea and studies were carried out to characterized chickpea-nematode interactions, to describe geographical distributions, general symptoms even at histopathological levels (Figures 3, 4 and Table 3). The two PPNs largest groups most represented in the world's agro-ecosystem (Hussey, 1989) with interesting infections trategies and life cycles (Figure 5) are CNs and RKNs (Figure 5). Although three CN species of *Heterodera* have been found associated with chickpea worldwide (Castillo et al., 2008), *Heterodera ciceri* (Figure 4) is the only nematode that can lead to significant economic loss. In semi-arid areas of cultivation, the eggs do not undergo dormancy but hatch in the presence of chickpea root diffusates (exudates), where there are suitable soil moisture and temperature conditions of at least 10°C. Chickpea is highly susceptible to damage by *H. ciceri* and, therefore, efforts have been dedicated to search for potential sources of resistance to transfer them into genotypes of commercial varieties. However, resistant accessions have been identified only in *C. bijugum, C. pinnatifidum*, and *C. reticulatum*, and were deposited in the ICARDA genebank (Table 1) (Malhotra et al., 2002). *Meloidogyne arenaria, Meloidogyne incognita* (Figure 3), and *Meloidogyne javanica* are the RKN species that cause damage to chickpea. All three are typically found in areas with warm climatic conditions, and attack chickpea especially in the Indian subcontinent. On the other hand, *Meloidogyne artiellia* (Figure 4), being well-adapted to cool and wet conditions, is widely distributed in the Mediterranean region, including Italy (Castillo et al., 2008). Particularly *M. arenaria, M. incognita*, and *M. javanica* induce large galls in chickpea roots, whereas *M. artiellia* gives rise to very small galls surrounding the feeding sites (Vovlas et al., 2005) or no galls in the infected roots (Table 3). Ansari and co-authors (Ansari et al., 2004) screened more than 7,000 accessions of chickpea germplasm for resistance to *M. javanica* (Treub) Chitwood; four promising nematode-tolerant genotypes were found and conserved in the chickpea ICRISAT genebank (Table 1).

**Table 3:** Selection of PPNs, associated with chickpea (font: https://www.cabi.org).

**Family**	**Species**	**Generic and characteristic symptoms**	**Countries**	**References**
*Meloidogynidae*	*Meloidogyne incognita*, *Meloidogyne arenaria*, *Meloidogyne javanica*	Whole plant: early senescence; Leaves: abnormally colored and wilted Roots: galls, swollen and reduced root system	Indian Subcontinent	Ali and Sharma, 2003; Vovlas et al., 2005
	Meloidogyne artiellia	Root with small or absent galls and protruded adult female	Mediterranean Basin	Vovlas et al., 2005
*Heteroderidae*	*Heterodera goettingiana*	Whole plant: stunted Leaves: pale green at an early stage, later chlorotic. Reduced number of flowers and pods, small or no seeds Roots: poorly developed, lacking nitrogen-fixation nodules.	North Africa	Di Vito et al., 1994
	*Heterodera ciceri*	Soil infestation in small circular area that should extend to entire field. Eggs don't undergo dormancy.	Turkey, Syria	Greco et al., 1988; Castillo et al., 2008
*Pratylenchidae*	*Pratylenchus thornei*	Whole plant: dwarfing distributed in patches Leaves: chlorosis and reduction shoot weight Roots: necrotic streaks or lesions, soft rot of cortex	Australia, India, Mexico, North Africa, Spain	Castillo et al., 1996
	*Pratylenchus penetrans*	Whole plant: reduced crop yield Leaves: chlorotic (pale, yellowing) Roots: may be thin, and with a reduced number of lateral roots.	North Africa, Spain, Turkey	Di Vito et al., 1994
*Hoplolaimidae*	*Rotylenchus reniformis*	Whole plant: distorted Leaves: abnormal colors Stems: stunting or rosetting Roots: external feeding and reduced root system	India, Egypt, Ghana	Mahapatra and Pahdi, 1996

## Genomics of *C. arietinum* L.: host response to pathogens and new applications of biotechnology

The chickpea genome has recently been released by two research groups (Jain et al., 2013; Varshney et al., 2013) and further analyzed (Parween et al., 2015; Thudi et al., 2016). The availability of a rich genomic platform of chickpea and its relatives, such as *C. reticulatum* (a source of interesting characteristics) (Gupta et al., 2017), provides insight into both genome diversity and domestication and therefore should be considered as a resource to improve chickpea resistance against biotic and abiotic stress.

**Figure 6:**
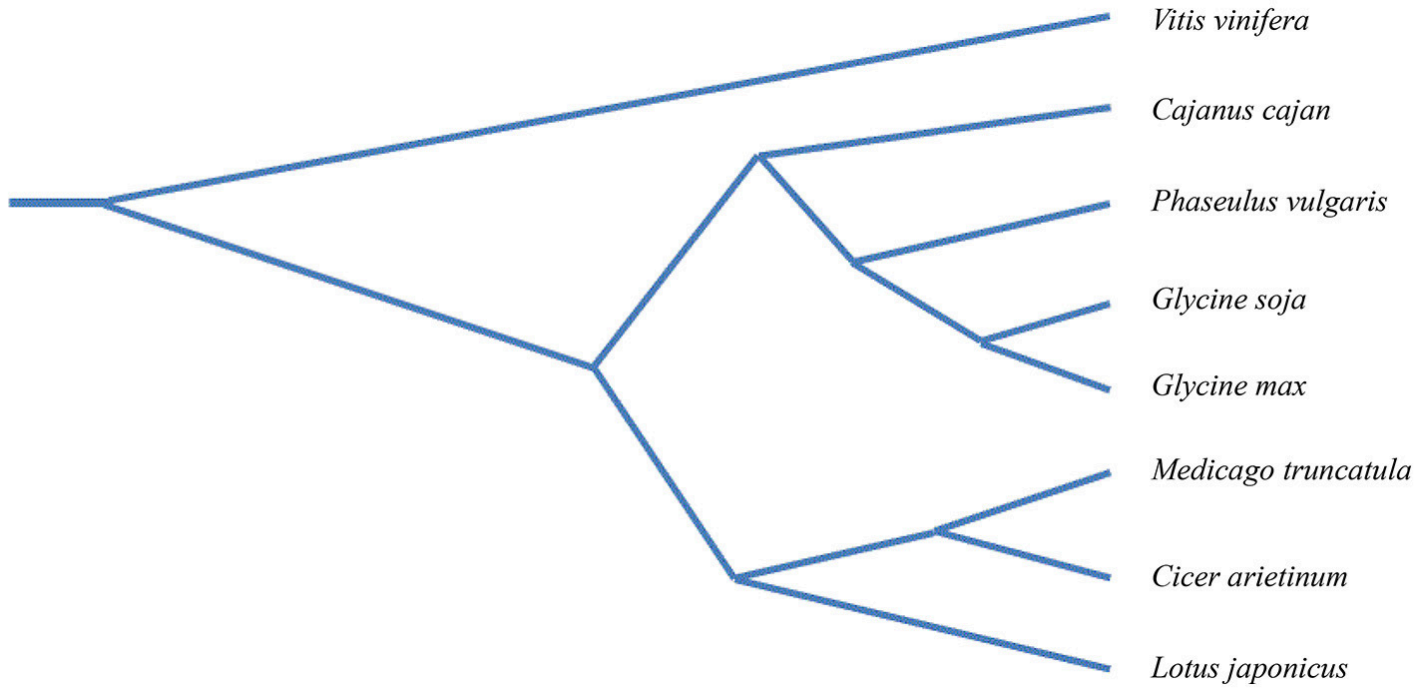
Phylogenetic tree of seven legume species with *Vitis vinifera* as the out-group. The phylogenetic tree was constructed with a genome-wide single-copy orthologous genes of legume species i.e., *Glycine max*, (cultivated soybean), *Glycine soja* (wild soybean), *Medicago truncatula* (barrel clover), *Lotus japonicus* (bird's-foot trefoil), *Cajanus cajan* (Pigeonpea) *Cicer arietinum* (chickpea), *Phaseolus vulgaris* (common bean). Modified from Zheng et al. (2016).

One of the most recurrent themes in plant pathology research is the highly adaptable nature of pathogens, including viruses and nematodes. These organisms possess the ability to harness and modify cellular resources in order to coexist with the plant host. Current genomics in legumes make it possible to study specific layers of plant-pathogen interactions directly using crop plants, including chickpea. A phylogenetic analysis of legume species constructed with genome-wide, single-copy orthologous genes shows that the closest relative to chickpea is *Medicago truncatula*, and secondarily *Glycine max* (Zheng et al., 2016) (Figure 6). *M. truncatula* and *G. max* are widely considered as model legumes and, therefore, studies in chickpea could benefit from those carried out in the model relative species. The availability of a genomic platform of the chickpea, together with recent advances in understanding the mechanisms of immune responses to plant pathogens, presents interesting perspectives for attenuating the damage caused in chickpea by biotic stress. Below we highlight the promising main topics.

## Plant immune defense response, effector targets, and RNA silencing in pathogen attack

**Figure 7:**
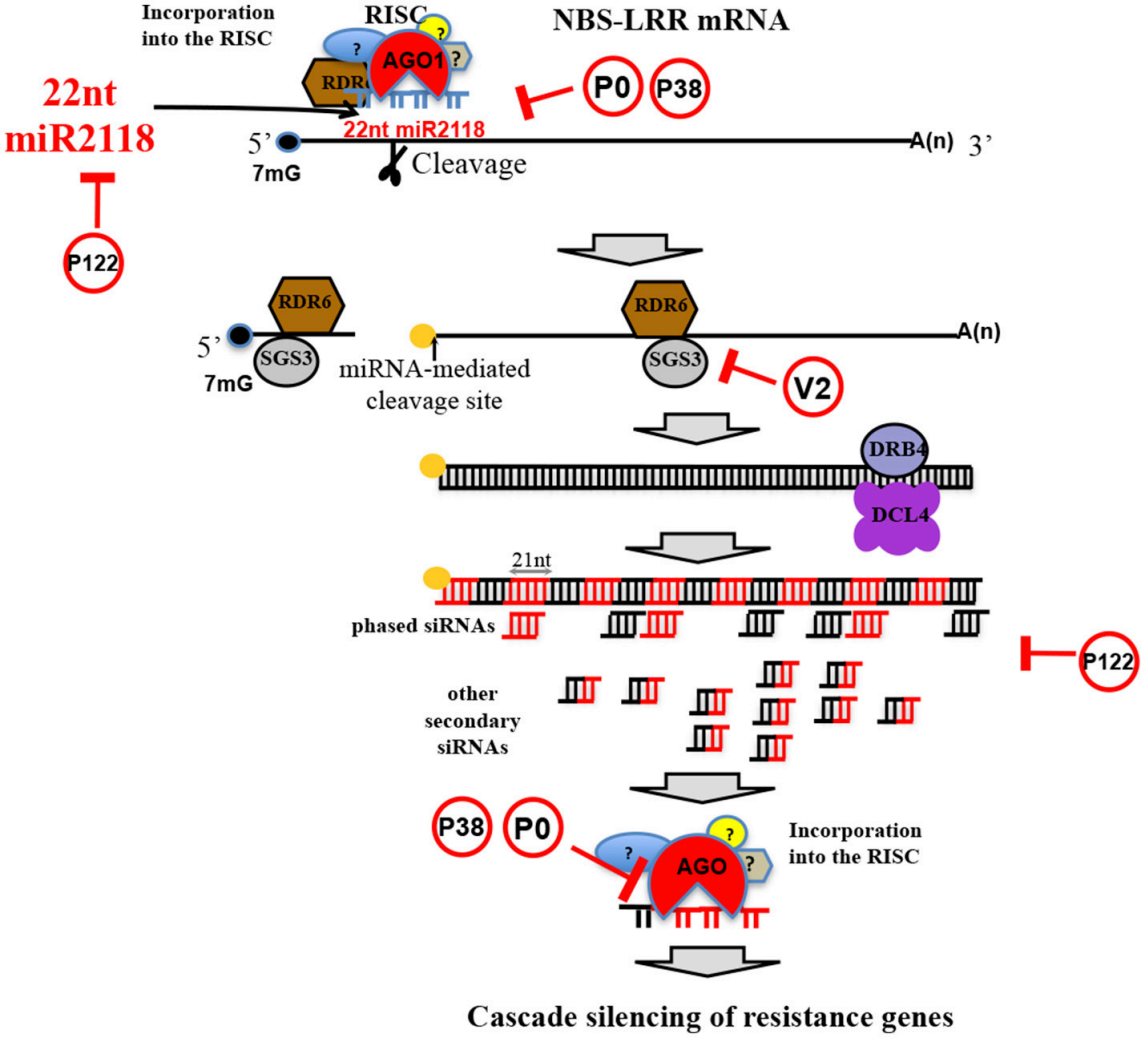
NBS-LRR silencing cascade mechanism. Schematic representation of NBS-LRR silencing cascade mechanism triggered by miR2118 (a legume specific miRNAs discovered in soybean), highly conserved in *C. arietinum*. In red circles, viral silencing suppressors (Csorba et al., 2015) that can impair the cascade mechanism.

Plants recognize pathogens and microbes through pathogen/microbe-associated molecular patterns (P/MAMPs). PAMPs are evolutionarily conserved molecules across kingdoms; in plants they carry out critical functions against several microbial attacks (Boutrot and Zipfel, 2017), including invasion of viruses, bacteria, fungi, and nematodes. For instance, it is widely accepted that the early stages of pathogen attack could be considered the key target step in plant defense strategies; this idea has also been recently extended to nematode parasitism (Holbein et al., 2016). PAMPs activate host defense responses (PAMP-triggered immunity or PTI) through a complex signaling cascade. Effectors should interfere with PTI responses, thereby leading to effector-triggered susceptibility (ETS). Manosalva and coauthors (Manosalva et al., 2015) showed that PPNs secrete conserved pheromones named “ascarosides,” eliciting MAMP response in various plants, and are exclusively identified in the phylum Nematoda. In turn, microbial virulent pathogens are able to overcome plant defense mechanisms by secreting effectors into the host. An effector protein can also be the elicitor of effector-triggered immunity (ETI) (Mandadi and Scholthof, 2013). If this first defense system is defeated, then plant resistance initiates a second mechanism which is more amplified and faster than PTI and usually develops in a form of programmed cell death known as the hypersensitive response (HR), leading the infected host cell to apoptosis. In this second detection system level, plants are able to recognize pathogenic effectors through nucleotide-binding site leucine-rich repeat (NBS-LRR) proteins and are characterized by leucine-rich repeats (LRR) that give them binding specificity. In fact, among the largest gene families in plants deputed to play roles in response to a broad range of pests and pathogens is the *R*-gene family, which mainly includes NBS-LRR genes (Zheng et al., 2016). Chickpea contains at least 153 NBS-LRR homolog genes in eight chromosomes (Varshney et al., 2013). This number is considerably lower than the number of orthologs in other legume species (Jain et al., 2013). Once discovered in *M. truncatula* and *G. max*, the cascade regulation of NBS-LRRs triggered by micro (mi)RNAs of the miR2118/482 superfamily members has been associated with nodulation events (plant-rhizobium interactions) and not to better specified plant pathogen defense strategies (Zhai et al., 2011). The interaction between miRNA and *R*-genes might have long-term evolutionary benefits by buffering NBS-LRR levels to reduce the fitness cost of these genes (Zheng et al., 2016). More recently, NBS-LRR secondary siRNA cascade mechanisms have been revealed to spawn valuable layers of non-race-specific resistance against viral and bacterial pathogens (Shivaprasad et al., 2012) (Figure 7). This recently discovered plant strategy seems to be independent from either the NBS-LRR protein additive effect of expression or from the *R*-gene-to-pathogen gene interaction. In chickpea leaf/shoot/floral tissues, 22 nt-long miR2118 is fairly present and targets NBS-LRRs (Srivastava et al., 2015), and the secondary siRNA mechanism involved in cascade regulation of NBS-LRR is present as well. Importantly, NBS-LRR regulation can be subverted by plant viruses. RNA silencing in plants and insects can function as a defense mechanism against invading viruses, and viruses have evolved viral suppressors of RNA silencing (VSRs) to overcome the host defense (reviewed by Csorba et al., 2015). VSRs can act on various steps of the different silencing pathways and, thus, can have a profound impact on host endogenous RNA-silencing regulatory pathways, including the generation and function of plant endogenous siRNA, such as miRNAs and secondary siRNAs (Figure 7).

Chickpea seems to be a permissive host for many plant viruses that are considered capable of inducing pathogenesis in many plant species. All of the plant viruses families infecting chickpea (Table 2) are known to express VSRs, which, in turn, have been shown to subvert RNA silencing machinery. For instance, PEMV-1 expresses the P0 protein, which has been shown to destabilize AGO1 protein (Fusaro et al., 2012) and, therefore, could hinder the miR2118-triggered, NBS-LRR–mediated cascade mechanism of *R*-gene silencing at several stages (Figure 7). Similarly, but with a different mechanism, TCV P38 can alter AGO1 activity (Azevedo et al., 2010) (Figure 7). Tobamovirus replicase (i.e., P122/P126) is known to bind miRNA and siRNA, preventing their stabilization and incorporation into the RNA-induced silencing complex (Csorba et al., 2007; Vogler et al., 2007) (Figure 7). All these VSRs from viruses infecting chickpea can block downregulation of NBS-LRR and the downstream cascade mechanism, inducing overexpression of *R*-genes with a wider coverage against viral and other pathogens, despite the low number of *R*-genes in the chickpea genome.

Recently, it has become clear that silencing pathways also play an important role in other plant pathosystems, including the onset of nematode parasitism. Indeed, through a transgenic approach, it has been shown that VSRs can subvert host RNA silencing machinery and increase the susceptibility to nematode parasitism (Walsh et al., 2017).

RNA silencing approaches have also been exploited in plants to control PPNs, given that RNA silencing mechanisms are also conserved in nematodes (Fire et al., 1998). Double-stranded RNA (dsRNA) can be produced through engineered plants that have the ability to silence target genes in nematode body. The delivery of dsRNAs from plant to nematode occurs by the ingestion process and can trigger RNA interference (RNAi), resulting in the inactivation of targeted genes (Gheysen and Vanholme, 2007). Availability of a genomic platform of PPNs is a prerequisite to identify the nematode genes responsible for the interactions and run loss-of-function (Abad et al., 2008; Denver et al., 2016). This could lead, for example, to adopt strategies based on the manipulation of nematode-derived protein elicitor(s), molecules able to induce a PTI-like response (Mendy et al., 2017). The ETI defense response in plant-nematode interaction is relatively better investigated than PTI, and often involves an HR reaction due to the initiation of the two characteristic “feeding structures” induced in the root by sedentary endoparasitic nematodes (Goverse and Smant, 2014) (Figure 5). A noteworthy case is the HR that takes place in the induction of several individual “giant cells” in *Mi-1*-resistant tomato plants infected by RKN-infective second-stage juveniles (J2) (Figure 5). By contrast, the deterioration of the “*syncitium*” (composed of hundreds of fused root cells, induced by *H. glycines* in soybean genotypes harboring a natural resistance gene at the *Rgh1* locus, is not characterized by typical cell death. *Rgh1*-mediated resistance seems to involve the collapse of the feeding site by nuclear and cytoplasmic fragmentation.

**Figure 8:**
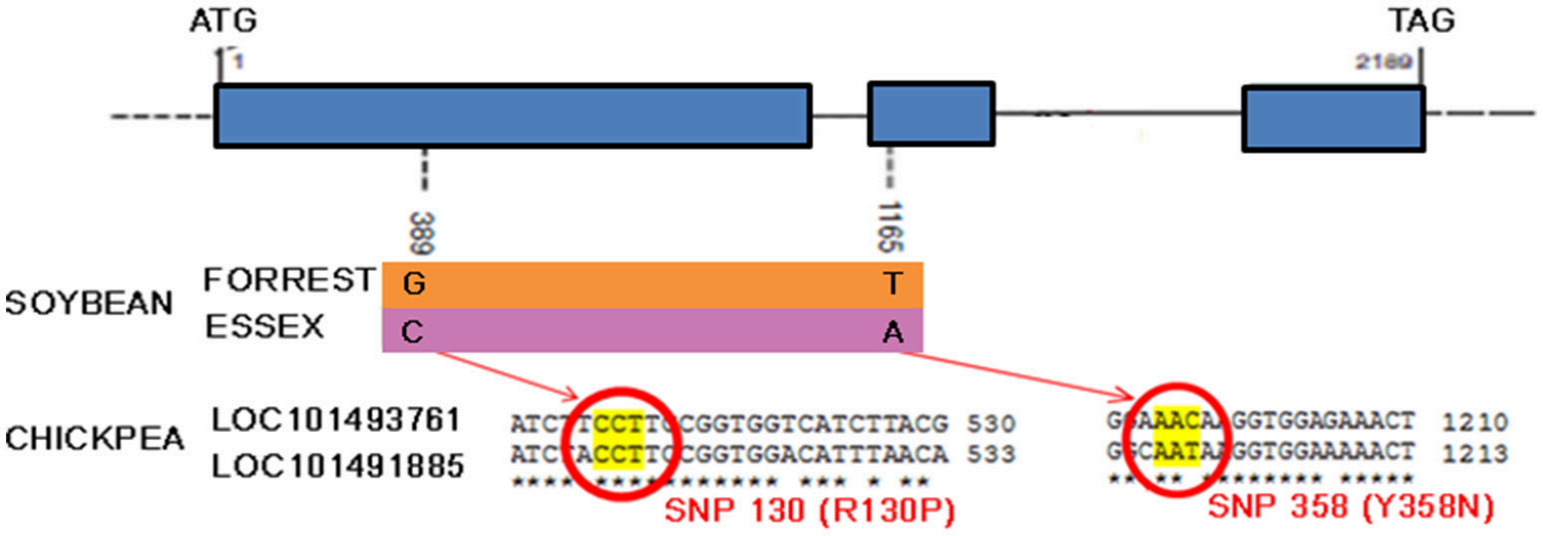
Serine hydroxymethyltransferase gene model. Shmt model and polymorphism in resistant (Forrest) and susceptible (Essex) soybean cultivars and alignment of predicted chickpea shmt 1-like mRNA sequences (NCBI reference XM_004504310.1 and XM_004502186.1) showing the two functional SNPs positions.

Recently, a novel and unique mechanism of plant resistance has been discovered through mutation analysis, gene silencing and transgenic complementation in soybean–*H. glycines* interactions. Wu et al. (2016) have demonstrated that the single dominant *Rgh4* locus, which is a major quantitative trait locus encoding serine hydroxymethyltransferase (SHMT), confers resistance to CNs (Wu et al., 2016). SHMT is an enzyme that is ubiquitous in nature and structurally conserved across kingdoms. The resistant allele possesses two functional single nucleotide polymorphisms (SNPs, denoted as P130R and N358Y) compared to that of the sensitive allele, rhg4. These mutations affect the kinetic activity of SHMT, which could result in folate deficiency inside syncytia and a nutritional deficiency that starves the nematode. This is a novel plant defense strategy against roundworm that could readily be extended to other important crops. Preliminary exploration within the chickpea genome has confirmed the presence of at least two *shmt* loci (Figure 8). These findings will likely boost research to extend the use of SHMT resistance to chickpea by identifying the source of positive functional SNPs in ancient local varieties or, alternatively, to apply novel technologies such as genome editing of functional SNPs.

## Biotechnology applications: current status on the genetic transformation of *Cicer arietinum* L.

The enhancement of tolerance to biotic and abiotic stress in chickpea can significantly increase its yield potential. However, due to the limited genetic pool, cross compatibility and lack of resistance sources to biotic constraints in the available germplasm the improvement of chickpea by means of conventional breeding faces serious limitations. Modern plant biotechnology tools offer several possibilities to finally overcome these limitations. The main objectives are (i) to enhance chickpea resistance to pests and other biotic and abiotic stress, and (ii) to achieve more sustainable food production in developing countries, such as in the semi-arid tropics where agrochemicals are inaccessible to low-income farmers (Sharma and Ortiz, 2000; Sharma et al., 2001). Most biotechnology approaches require skills and tools for manipulating the genome of a plant, either through transgenics or other means, and the process always includes plant transformation and regeneration steps. Chickpea, like other large-seed grain legumes such as faba bean, pigeonpea, and common bean, is considered to be “reluctant” to *in vitro* transformation and regeneration (Somers et al., 2003). Therefore, one critical point of chickpea productivity improvement remains the development of reliable genetic transformation techniques.

Plant genetic transformation is defined as the method for the delivery, integration, and expression of foreign genes into plant genomes (Atif et al., 2013). There are two main methods that enable delivery of the gene into the plant genome: (i) direct gene transfer (mediated by physical or chemical forces) and (ii) *Agrobacterium*-mediated genetic transformation, where *Agrobacterium tumefaciens* is used as a vehicle to introduce foreign genes into the plant genome.

In the case of the chickpea, many research efforts were undertaken to improve resistance against major biotic stress, such as pod borers (*Heliothis armigera* [Hub.]), aphids (*Aphis craccivora*), bruchids, fungal diseases (*Fusarium oxysporium/F. udum*), and abiotic (drought and salinity) stress, as well as the nutritional quality by increasing the sulfur-containing amino acid content. The first transformation studies with chickpea were performed by Srinivasan et al. (1991) and Islam et al. (1994) using callus culture; shoot regeneration was not possible. Although these studies were unsuccessful due to poor regeneration, they showed the susceptibility of chickpea to infection with *A. tumefaciens* and proved its potential as a transformation vector for chickpea. Afterwards, generation of transgenic chickpea was reported with varying degrees of success. To our knowledge, however, the number of reports describing the successful production of transgenic chickpea using either *Agrobacterium*-mediated or particle bombardment transformation is still very limited (Mishra et al., 2012; Atif et al., 2013; Tripathi et al., 2013). Table 4 summarizes chickpea transformation studies. Most of the first attempts on genetic transformation used the *Agrobacterium*-mediated method with few exceptions, where particle bombardment was employed (Tewari-Singh et al., 2004; Ganguly et al., 2014). Indurker et al. (2007) reported a successful transformation protocol (16% transformation frequency) using particle bombardment with gold particles as micro-carrier, in combination with helium pressure of 900 psi on epicotyl explants of the cultivars ICCC37 and PG-12. The construct was a pHS102 plasmid harboring the reporter gene *uidA*, neomycin phosphotransferase II (*nptII*) and insecticidal *cry1Ac*. Fontana and colleagues (Fontana et al., 1993) reported the first successful chickpea transformation protocol after transformation of embryonic axes with *A. tumefaciens*. The transferred genes were successfully inherited into subsequent generations. Molecular evidence for the transgenic nature was confirmed by studying the integration and expression of β-D-glucuronidase and *nptII* genes as well as the integration and expression of the transferred genes. Later, other reports described new protocols (Krishnamurthy et al., 2000; Polowick et al., 2004; Sarmah et al., 2004; Senthil et al., 2004; Sanyal et al., 2005) improved for their simplicity and relatively short time required to produce transgenic plants (T0) without the callus phase (4–6 months). From surveying the literature on chickpea transformation, it can be concluded that the average frequency of *Agrobacterium*-mediated transformation is 0.1–5.1%, which is very low compared to model plants such as *G. max* and *M. truncatula* (96 and 80%, respectively) (Iantcheva et al., 2001; Li et al., 2017). However, with the ICC10943 cultivar and using sonication-assisted, *Agrobacterium*-mediated transformation (SAAT) cases of transformation efficiency higher that 25% have been reported (Bhattacharjee et al., 2010; Table 4). Therefore a wider utilization of SAAT for chickpea transformation can be foreseen, which should be nonetheless tested on several other varieties.

**Table 4:** Genetic transformation studies in chickpea.

**Transformation method**	**Cultivar**	**Explant type**	**Transferred genes**	**Transformation frequency %**	**References**
*Agrobacterium*-mediated transformation	Local ecotype	EAx	*uid*A, *npt*II	4^*^	Fontana et al., 1993
	ICCV1, ICCV6 and desi (local) variety	EAx	*uid*A, *npt*lI	e.g., ICCV-6: 1.96	Kar et al., 1996
	PG1, PG12, Chafa and Turkey	EAx	*uid*A, *npt*II	e.g., Turkey < 1.5	Krishnamurthy et al., 2000
	H-208, ICCL87322, K-850, Annigiri, and ICCV5	EAx	*uid*A, *bar*	5.1^§^	Senthil et al., 2004
	Semsen	Halved EAx attached to cotyledon	*nptII*, bean α*AI1*	0.72^¢^	Sarmah et al., 2004
	CDC Yuma	EAx	*uid*A, *npt*II	3.1^∧^	Polowick et al., 2004
	C-235, BG-256, Pusa-362 and Pusa-372	Pre-conditioned CNs	*cry*1Ac, *npt*II	e.g., BG 256: 1.12	Sanyal et al., 2005
	K-850	EAx	α-*ai, uid*A, *npt*II	0.3	Ignacimuthu and Prakash, 2006
Sonication-assisted *Agrobacterium* mediated transformation (SAAT)	ICC10943 and ICC10386	Decapitated embryo	*uid*A, *hpt* II	ICC 10943: 26 ± 2, ICC 10386: 24 ± 3^Δ^	Pathak and Hamzah, 2008
	ICCV89314	Single cotyledon with half embryo	ASAL, *nptII, gusA*	0.066 ± 0.003 (mean ± SE)	Chakraborti et al., 2009
	C-235, Annigiri and K-850	Wounded apical dome of shoot apex	*uid*A, *bar*	2.43	Singh et al., 2009
	C-235	EAx with half portions of both cotyledons	*pmi*	3	Patil et al., 2009
	C-235	AMEs	P_5_CSF_129_A, *npt* II, *uid*A	Not mentioned	Bhatnagar-Mathur et al., 2009
	Semsen, ICCV89314	Cotyledon with half EAx	*cry*2Aa, *npt*II	0.3	Acharjee et al., 2010
	Pusa-256, KWR-108, Pusa-1003 and local line (from market)	Cotyledon- and cotyledonary-node-derived-callus and EAx	*uid*A, *hpt*	e.g., KWR 108: 23.45	Bhattacharjee et al., 2010
	Annigeri	CNs	*P5CS, hpt, uid*A	Not mentioned	Ghanti et al., 2011
	P-362	CNs	*cry*1Ab, *cry*1Ac, *npt*II	2.77	Mehrotra et al., 2010
	C-235, BG-256, P-362 and P-372	Immature cotyledons, EAx	*uid*A, *npt*II	e.g., P 362: 2.08	Tripathi et al., 2013
	C-235	AMEs	DREB1A, *npt*II	Not mentioned	Anbazhagan et al., 2015
	DCP-923	EAx	fused *cry1*Ab/Ac, *hpt*	Not mentioned	Ganguly et al., 2014
	P-362	CNs explants	*nptII, uid*A, modified human α_1_-PI, *cry1A*b, *cry1Ac*	Not mentioned	Yadav et al., 2017
	C-235	AMEs explants	*uid*A, *npt*II	1.2	Srivastava et al., 2017
Particle bombardment	ICCV1, ICCV6	EAx	*npt*II, *cry*1Ac	Not mentioned	Kar et al., 1997
	P-362, P-1042 and P-1043	Decapitated embryo	*pat, nptII, uid*A, *AK*	Not mentioned	Tewari-Singh et al., 2004
	Chaffa, PG12, ICCC37 and ICCC32	EAx, epicotyl and stem	*npt*II, *uid*A, *cry*1Ac	16 ± 0.33^£^	Indurker et al., 2007

*EAx, embryonic axis; AMEs, Axillary meristem explants; CNs, Cotyledonary Nodes; uidA, β-Glucuronidase, commonly referred to as the gus gene; nptII, neomycin phospho transferase II; bar, Basta (bialaphos) resistance; αAI1, bean-α amylase inhibitor 1; pat, phosphinothricin-acetyltransferase; AK aspartate kinase; cry1Ac, insecticidal crystal toxins 1Ac; α-ai, α-amylase inhibitor; ASAL, Allium sativum leaf agglutinin; gusA, β-Glucuronidase; pmi, phosphomannose isomerase; P_5_CSF_129_A, D1- pyrroline-5-carboxylate synthetase F_129_A; cry2Aa, insecticidal crystal toxins 2Aa; hpt, hygromycin phosphotransferase; P5CS, pyrroline-5-carboxylate; DREB1A, dehydration response element B1A; α_1_-PI, modified human Alpha-1-proteinase inhibitor*.

* *Number of whole plants transformed/initial number of embryos*;

§ *Number of confirmed independent lines/number of initial seeds*;

¢ *18 independently derived transgenic plants obtained from a total of 2,500 explants (explant that consisted of one cotyledon attached to half embryonic axis)*;

∧ *7 separate experiments with the use of shoot elongation media (MS)*;

Δ *Obtained by dividing [100 times the number of confirmed transformed plants of independent lines (both PCR and Southern blot positive)] by the number of treated explants*;

£ *Epicotyl, average of three experiments with 150 explants each*.

## Perspectives

The strong potential of genetic transformation techniques for crop improvement is unquestionable. The clustered regularly interspaced short palindromic repeat (CRISPR)-associated protein 9 (Cas9) DNA editing system has recently been developed as a new method for genome engineering. It is based on the type II CRISPR-associated immune system that protects bacteria against invading DNA viruses and/or plasmids (Jinek et al., 2012). Genome editing by CRISPR/Cas9 as well as other techniques, including zinc finger nucleases (ZFNs) and transcription activator-like effector nucleases (TALENs), have been applied to edit the genome in several plant species (Kim and Kim, 2014). The successful utilization of CRISPR/Cas9-directed genome editing in plant species has been reported and also includes the two relatives, i.e., the legume models *G. max* and *M. truncatula* (Li et al., 2015; Meng et al., 2017). CRISPR/Cas9 gene editing technology is currently revolutionizing genetic studies and crop improvement because it can be applied with high-throughput and at genome-scale (Yang et al., 2017). To our knowledge, no research effort has been made to implement this system in chickpea. The application of CRISP/Cas9 in chickpea genome editing will not only provide answers to basic biological questions but will also reduce public concern about transgenic plants, owing to its non-GMC nature. In most cases, Cas9 and guide (g)RNAs are delivered into plant cells by *Agrobacterium*-mediated T-DNA transformation or by physical means, such as PEG-mediated transformation of protoplasts or biolistic transformation of calluses. In the case of chickpea and other legumes, this approach could face limitations due to the difficulties of transformation and regeneration from callus. An alternative approach that could help to overcome these limitations is the identification and engineering of plant viruses as a tool for systemic gene editing in plants. Some successful examples are already available. Cabbage leaf curl virus, a geminivirus, is able to deliver gRNA and induce systemic gene mutations in plants (Yin et al., 2015). An RNA viral vector based on Tobacco rattle virus (TRV) has been demonstrated to serve as a vehicle to deliver genome-engineering reagents to all plant parts, including meristems. This provides a general method for easily recovering seeds with the desired modifications, obviating the need for transformation and/or tissue culture (Ali et al., 2015). More recently a legume virus, the Pea early-browning virus, has been demonstrated to be more efficient than TRV for these applications (Ali et al., 2017). An additional challenge would be the identification of the best DNA or RNA viruses able to fully infect chickpea to be engineered and used as viral vectors dedicated to genome editing.

Genome editing of chickpea may help improving specific characteristics of a crop with limited genetic pool and lack of resistance sources. An emblematic case would be the modification of functional SNPs in the SHMT gene (Figure 8) in order to confer resistance to nematodes or to modify miRNA target sites in NBS-LRR genes (Figure 7), ensuring the up-regulation of certain functional *R*-genes.

Perspectives for the improvement of chickpea should also take into account the genomic selection approach. It facilitates the rapid selection of superior genotypes and accelerates the breeding cycle and it has been applied with a large success in many other crops (Crossa et al., 2017).

Chickpea cultivations may constitute a reservoir of viral entities. Indeed, chickpea seems to be a permissive host for many viruses and viroids, thus ensuring their maintenance in agro-ecosystems: most of the hosted viruses are symptomless in chickpea, but pathogenic for other plant species. Viral metagenomics is currently the tool most indicated for surveys of virus-infected plants. In addition, metagenomics approaches can help to discover novel infectious entities and microbes either hosted by or associated to chickpea. This could help scientists better identify and describe the multi-trophic interactions that may influence nematode reproduction or plant-rhizobia interactions.

To modulate plant PPNs, several transgenic strategies have been used, such as (i) cloning of resistance genes from natural resources and transfer to other plant species; (ii) overexpression of different protease inhibitors; and (iii) suppression of nematode effectors in plants using RNAi (Ali et al., 2017). RNAi (Rosso et al., 2009; Banerjee et al., 2017) is worth exploring more in depth, particularly RNAi-based technology combined with peptide expression which disrupt nematode sensory activities (Fosu-Nyarko and Jones, 2015). Moreover, a number of CRISPR/Cas9 genome-editing protocols have been established in *Caenorhabditis elegans* (Friedland et al., 2013; Zamanian and Andersen, 2016). Genome manipulation with novel developments in this model organism, research, and advances in parasite genomics could open new doors to the biology of closely related nematode parasites during their interaction with legumes. A more in-depth understanding of the potentiality in biotechnologies for legume pest management will at least modernize chickpea breeding programs, targeting a greater impact on food and nutrition security, climate change adaptation and worldwide diffusion.

## Author contributions

All the authors have contributed to the review with their proper specific expertise in plant science, plant pathology, and plant biotechnology.

### Conflict of interest statement

The authors declare that the research was conducted in the absence of any commercial or financial relationships that could be construed as a potential conflict of interest.
